# Use of Ethnomedicinal Plants by the People Living around Indus River

**DOI:** 10.1155/2014/212634

**Published:** 2014-03-20

**Authors:** Sakina Mussarat, Nasser M. AbdEl-Salam, Akash Tariq, Sultan Mehmood Wazir, Riaz Ullah, Muhammad Adnan

**Affiliations:** ^1^Department of Botany, Kohat University of Science and Technology, Kohat-26000, Pakistan; ^2^Arriyadh Community College, King Saud University, Arriyadh-11437, Saudi Arabia; ^3^Department of Botany, University of Science and Technology, Bannu-28100, Pakistan; ^4^Department of Chemistry, Government College Ara Khel, FR Kohat-26000, Khyber Pakhtunkhwa, Pakistan

## Abstract

The objective of present study was to document and preserve ethnomedicinal knowledge use to treat different human ailments by traditional healers of Dera Ismail Khan region, Pakistan. Field work was conducted between February 2012 and January 2013 using semistructured questionnaires. Data was collected from 120 traditional healers through questionnaire survey. Traditional healers in the study area use 70 plant species mostly herbs (57%) for ethnomedicinal and other purposes. The highest F_IC_ values (0.80) were obtained each for gastrointestinal and kidney problems followed by respiratory infections (0.72) and skin infections (0.73). There was a significant correlation (*r* ^2^ = 0.950; *p* < 0.01) between the age and traditional knowledge of respondent. Direct matrix ranking indicated *Morus alba* and *Dalbergia sissoo* as highly multipurpose and threatened species in the study area. The results showed high dependency of local inhabitants on medicinal plants in meeting their primary health care needs. Moreover, the traditional knowledge has been restricted to elder people. Protection measures should be taken in order to conserve precious multipurpose species that are facing overexploitation. Medicinal plants treating major ailments in the region may be subjected to phytochemical and pharmacological investigations for the identification of bioactive compounds.

## 1. Introduction

Medicinal plants have important contributions in the healthcare system of local communities as the main source of medicine for the majority of the rural population [1]. Out of the total 422,000 flowering plants reported from the world, more than 50,000 are used for medicinal purposes [2]. About 60% of the world population and 80% of the population of developing countries rely on traditional medicine. According to Bhat et al. [3], more than 4.5 billion people in the developing world rely on medicinal plants as components of their healthcare. The highest popularity of medicinal plant in rural areas is due to high cost of allopathic drugs and side effects [4].

In the early 1950s, up to 84% of Pakistani population was dependent on indigenous medicines for traditional health practices [5], but now this is practiced only in the remote rural areas [6]. Due to modernization, people are getting far from this treasure and this knowledge is eroding at a much faster rate [7]. Ethnobotanical studies in various areas of Pakistan have been carried out [8–12]. It is believed that such studies can constitute the starting point for the development of new drugs and useful substances [13].

The present study was aimed at investigating the traditional utilization of plants of Dera Ismail Khan District located in the north-west region of Pakistan. The study area is the part of the country's richest biodiversity centre and a source of ethnobotanical knowledge. Very few ethnobotanical studies have been conducted in this region. The main objectives of the present study were (i) to identify and explore plant species that are used locally for the treatment and prevention of various diseases, (ii) to document traditional recipes from medicinal plants including methods of preparation, dosage, and modes of administration, (iii) to select candidate medicinal plant species of high priority for phytochemical and pharmacological analyses in our subsequent studies, and (iv) to assess the plants conservation issues of the study area.

## 2. Materials and Methods

### 2.1. Study Area

**Figure 1:**
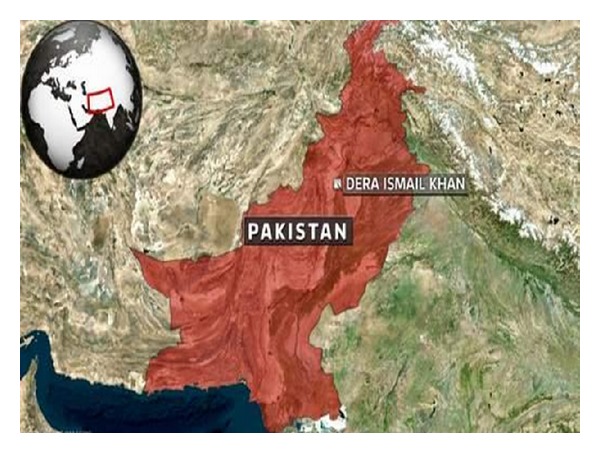
Map of the study area.

The present study was carried out in the Dera Ismail Khan often abbreviated to D. I. Khan, which is a city in Khyber Pakhtunkhwa province, Pakistan (Figure 1). D. I. Khan is with an area 7326 km^2^ and is situated between 31°.15′ and 32°.32′N latitude and between 70°.11′ and 71°.20′E longitude [14]. Most of the area of the district consists of flat dry alluvial plain, commonly known as Daman, which makes up more than 80 percent of the area where a large number of streams and hill torrents discharge water [15]. D. I. Khan supports xerophytic and aquatic vegetation and their associated species of wild fauna [16]. Dominant plant species are* Acacia modesta, Acacia nilotica, Calotropis procera, Morus alba, *and* Eucalyptus camaldulensis. *The maximum and minimum mean temperature recorded during June is from 42°C to 27°C, while in winter season the minimum temperature recorded is 20°C and maximum temperature is 40°C [17]. Precipitation mainly falls in two distinct periods: in the late winter and early spring from February to April and in the monsoon in June and July. One of the most famous products of this district is the “Village Dhakki date,” which is exported to the Middle East, United States, and Europe. This district also produces wheat, sugar cane, rice, and a famous variety of mangos. Most of the population of the area is rural with low literacy rate and they also lack modern health facilities; hence, they are more dependent upon natural resources especially plants for their healthcare and to compensate their low income as well.

### 2.2. Data Collection

Field work was carried out between February 2012 and January 2013. A total of eight field visits were made in four different seasons for data collection. Each visit lasted over 20 days in the field. A total of 120 informants were selected on the basis of information provided by the local administrator and elder people of the study region. Ethnic groups including* Marwat*,* Lodhi, *and* Sial *inhabit the study area.* Marwat *and* Lodhi *are more concentrated and are generally more aware of the traditional knowledge. The selected healers were well known in the community due to their long practice in service provision related to traditional health care. The informants were native-born or had been living in the study area for a long time. Prior to data collection, group meeting was held with the help of village's head in order to explain to the informants (i) theme of present study and (ii) assurance that their knowledge would be a great contribution in conserving the indigenous knowledge of the area. Prior to survey, a semistructured questionnaire was designed and pretested with five informants to find out its suitability for the present study and later on modified according to response of informants. The revised questionnaire was used for gathering data from individual informant about medicinal plants of the study area. The questionnaire contained no strict questions and informants were allowed to speak spontaneously and without pressure. Our final purpose was to obtain the complete list of medicinal plants used and/or known by each informant. All interviews were carried out in local language (*Saraiki*) of the study area. In addition, a total of four focus group discussions with 30 informants in each group were also designed to gain further information on medicinal plants at the community level and to prove the reliability of data collected through semistructured interviews [18]. Questionnaires designed to the respondents (traditional healers) about medicinal plants knowledge were mainly focused on local name of a particular medicinal plant, types of disease treated, mode and method of remedy preparation, parts of the plants used, use of fresh or dry plant parts, use of single or mixture of plants for remedy preparation, mode of administration, dose requirement, and usable duration regarding each medicine. Questionnaires also contained questions regarding sociocultural information.

### 2.3. Medicinal Plants Collection and Preservation

Plant samples were collected from the field and were dried and compressed in newspapers. Newspapers were changed daily until they remained dry after compression. Identification of plants was done by the expert taxonomists Dr. Waheed Murad and Dr. Azizullah of Kohat University of Science and Technology, Kohat, Pakistan. Scientific names, family names, and publication authors were corrected according to the flora of Pakistan and software index kewensis [19]. Pressed plant samples, plant photographs, and descriptions were assigned voucher numbers and deposited at the herbarium at the Department of Botany, Kohat University of Science and Technology, Kohat, Pakistan.

### 2.4. Data Organization

Data was organized and analyzed using Microsoft Excel software. The habits of the plants were categorized into three groups, that is, herbs, shrubs, and trees, using available literature [19]. The status of recorded plants was divided into three groups of wild, cultivated, and both wild and cultivated. The parts used by the healers were categorized into 11 groups, that is, fruit, leaves, whole plant, seeds, bark, root, and so forth. Human ailments treated by the traditional healers were divided into 11 categories such as gastrointestinal infection, respiratory infection, and fever. Route of administration of plant remedies was classified into three groups such as oral, dermal, and both oral/dermal and nasal. Questionnaire data was analyzed for basic categorization of the respondents' gender, age groups, literacy ratio, and occupation.

### 2.5. Data Analysis

#### 2.5.1. Informant Consensus Factor (F_IC_)

Descriptive statistics were used to examine and summarize the ethnobotanical data. Based on the information obtained from the informants, the ailments reported were grouped into a total of 11 categories. The F_IC_ results could be useful in prioritizing medicinal plants for further scientific validation of plants and plant products [20, 21], as pharmacologically effective remedies are expected from plants with higher F_IC_ values [22]. The informant consensus factor (F_IC_) was calculated to estimate user variability of medicinal plants [23, 24]. F_IC_ values range from 0.00 to 1.00. High F_IC_ values are obtained when only one or a few plant species are reported to be used by a high proportion of informants to treat a particular ailment, whereas low F_IC_ values indicate that informants disagree over which plant to use [23]. High F_IC_ values can thus be used to pinpoint particularly interesting species for the search of bioactive compounds [24]. F_IC_ is calculated using the following formula:

(1) $$\begin{array}{c} {\text{F}}_{\text{IC}}=N_{\text{ur}}-\frac{N_{\text{t}}}{\left(N_{\text{ur}}-1\right)}, \end{array}$$

where *N*_ur_ is the number of individual plant use reports for a particular illness category and *N*_t_ is the total number of species used by all informants for this illness category.

### 2.6. Direct Matrix Ranking (DMR)

Data on use diversity of multipurpose medicinal plants were evaluated by direct matrix ranking (DMR) exercises as described in Cotton [25] that involved fifteen (ten men and five women) key informants. Participants for this exercise were selected based on their long years of experience as traditional herbal practitioners in the study area as described in Yineger et al. [26].

#### 2.6.1. Pearson's Correlation

This statistical test was applied between the age of the respondents and number of plants known to them. The test was carried out using SPSS [27].

## 3. Results

**Figure 2:**
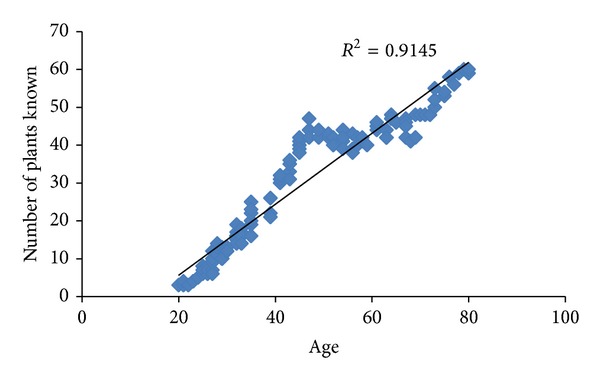
Comparison between age of the people and number of plants known (Pearson's correlation (*r* ^2^ = 0.950), *n* = 120).

Among the 120 informants, 50 (41.5%) were male and 70 (58.5%) were female. The largest proportion of the respondents was of the elderly, above 40 years old (Table 1). More than half of the respondents were illiterate (52.5%), whilst most of those with an education received merely primary education (25.8%) which reflects the unavailability of educational institution in the area (Table 1). Majority of females (90%) were housewives while 44% of males were farmers followed by 24% of shopkeepers. These very basic results also reflect the reality that indigenous knowledge is well established but seems to be decreasing in the younger generation. The indigenous knowledge showed a significant negative correlation (*r* = −0.95, *P* < 0.01) with the age of the respondents (both male and female) (Figure 2).

The present study provides information of ethnomedicinal uses of 70 plant species belonging to 39 families and 62 genera (Table 2). Out of 39 families, the dominant family with highest number of medicinal plants was Solanaceae (5 species) followed by Moraceae, Poaceae (4 species), and 3 species each in Liliaceae and Asteraceae. Moreover, the local healers mostly use herbs (57%) followed by trees (29%) (Table 2). Of the 70 species, 50% were cultivated while (44%) were wild (Table 2).

**Figure 3:**
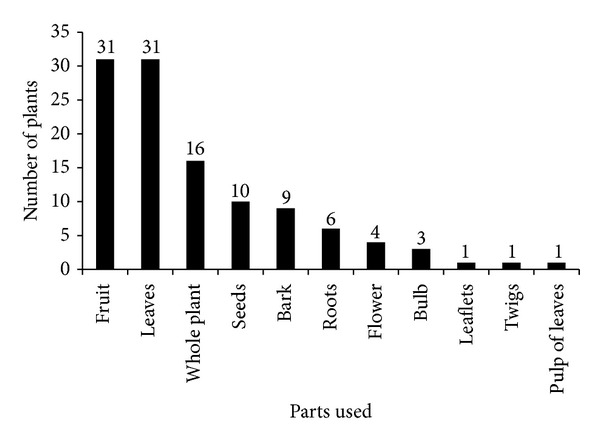
Medicinal plants part used.

Different parts of medicinal plants are used as medicine by the traditional healers (Figure 3). Among the different plant parts, the leaves and fruit (31%) are the most frequently used for the treatment of diseases followed by whole plant parts, roots, barks, tubers, seeds, and stems. Ethnomedicines were mostly taken through oral route (70%) followed by dermal/oral (15%) and dermal (11%). Decoction is the most common method used for remedy preparation (Table 2). The additives like milk, butter, boiled coffee, and food are commonly believed to serve as a vehicle to transport the remedies. The most commonly treated disease (34%) in the study area was gastrointestinal disorders (Table 3). The healers used usually fresh plant parts for the preparation of ethnomedicines (Table 2).

There is no standardized measure on the dose for most of the ethnomedicines in the study area. The dose depends on the traditional healer that prepares the herbs for medicinal purpose or it may also depend upon the disease severity. The dosage of certain plants in the region varied according to the type of illness ranging from two spoonfuls (e.g., for treatment of jaundice using syrup prepared from* Azadirachta indica*) to a cup or glass (e.g., for blood purification and abdominal pain stained water from fruit of* Withania coagulans*). Most of the ethnomedicines are prepared using single plant in the region while some others are prepared by the mixing parts of more than one plant; for example, fresh leaves of* Mentha viridis *and* Ocimum basilicum *and fruit of* Foeniculum vulgare *are mixed and boiled to make tea used for stomach problems, equal quantities of fruits of* Coriandrum sativum *and* Foeniculum vulgare *are mixed and crushed to make powder and used as carminative, and extract of bulb of* Allium cepa *and* Mentha viridis *is mixed and used for cholera.

About 11 disease categories were identified from the investigated region. The highest F_IC_ values were gastrointestinal (0.80), respiratory (0.72), skin infections (0.73) and kidney problems (0.80) (Table 3). The highest plant use citation was for gastrointestinal disorders (122) followed by kidney problems (37) and respiratory infections (23). The output of the DMR exercise on ten multipurpose medicinal plants enabled to identify which of the multipurpose plants is the most to be under pressure in the area and the corresponding factors that threaten the plant. Accordingly,* Morus alba *and* Dalbergia sissoo* ranked first (the most threatened);* Zizyphus jujuba *ranked second;* Tamarix aphylla *ranked third;* Withania coagulans *ranked fourth (Table 4). Results also indicated that those multipurpose medicinal plant species are currently exploited more for fodder, fuel, construction, and agricultural tools purposes besides their medicinal role.

## 4. Discussion

### 4.1. Medicinal Plants and Related Knowledge

The present study provides information on 70 medicinal plants used in the study area by local traditional healers. The study revealed that the people of the region have been using plant resources for their various ailments. The local people know the useful plants and preparation of recipes through personal experience and ancestral prescription and long utility [28].

Dominance of medicinal plant species from families of Solanaceae, Asteraceae, Poaceae, and Moraceae could be attributed to their wider distribution and abundance in the flora area [29, 30]. As leaves and fruits of medicinal plant species were reported to be harvested for most remedy preparations, gathering of medicine may have little negative impact on the species. It is well recognized by conservationists that medicinal plants primarily valued for their root parts and those which are intensively harvested for their bark often tend to be the most threatened by overexploitation [31]. Results also showed prominent use of freshly harvested plant parts for traditional remedy preparation used against various ailments. The recurrent use of freshly harvested medicinal plant materials in the area is reported to be related to the notion of attaining high efficacy using active ingredients of fresh plant parts which they thought could be lost on drying. Other ethnomedicinal inventories [26, 32] have also indicated wide use of fresh plant materials for remedy preparations due to reportedly better efficacy related factors than use of dried plant materials.

### 4.2. Growth Form and Status of Medicinal Plants

Present study elucidates that the herbs are the major growth form used in the region for curing human diseases followed by trees. A high usage of herbs in some studies could be an indication of their abundance, easy availability, and centuries-old traditional knowledge of the healers. The trend of using more of herbaceous plants could be advantageous as it is easier to cultivate them when they are short in supply. According to our study, most of the medicinal plants are being cultivated in the region. The high proportion of woody plants in our survey is likely associated with the ability of trees to withstand long dry seasons, thus resulting in their abundance and year-round availability in arid and semiarid areas. Thus the variation in medicinal plants growth form might be associated with different sociocultural beliefs, ecological status, and variation in practices of traditional healers of different regions or countries.

### 4.3. Preparation, Route of Administration, and Dosage of Medicinal Plants

The healers of the region mostly used ethnomedicines in decoction form. The medicinal plant decoctions for various ailments might be related to their proven effectiveness over many years of trial and indigenous knowledge accumulated on efficacy of such preparations. Additives (milk, butter, boiled coffee, and food) that are commonly believed to serve as a vehicle to transport the remedies are also necessary to minimize the bitterness, vomiting, and diarrhoea and to make the remedy more palatable. The finding is in line with other studies indicating that the oral route is the most preferred mode of administration [33, 34]. Preparation of plant medicines from several plant parts is believed to cure diseases more rapidly compared to single plant medicine [35].

### 4.4. Priority Medicinal Plant Species and the Ailments That They Treat

The highest number of plant species and highest F_IC_ value were reported for gastrointestinal, respiratory, kidney, and skin infections. This may be related to a high prevalence of these ailments. Gastrointestinal disorders and respiratory infections, particularly cholera, diarrhoea, dysentery, cough, asthma, and bronchitis, are a major concern not only in the study area but also in the whole country and result in high mortality rate if not treated promptly [36]. 

In our study the lowest F_IC_ value below 0.05 was only recorded for wound healing category, which would typically result from plant use to treat rare diseases; however all other diseases have F_IC_ value above 0.05, suggesting that our survey addressed medicinal plant species commonly used to treat common human ailments in the study areas. The high F_IC_ value medicinal plants contain variety of bioactive compounds and many of them have been scientifically proved by various studies. For example the natives of the region are using a large of number of plants like* Solanum nigrum*,* Calotropis procera*,* Grewia asiatica, Punica granatum*, and so forth for the treatment of diarrhoea, dysentery, and other gastrointestinal disorders, while many plants like* Eucalyptus camaldulensis, Coriandrum sativum, Datura metel*, and so forth are being used for respiratory diseases. The aforementioned plants contain variety of chemical constituents like tannins, saponins, alkaloids, flavonoids, and phenol compounds that are responsible for their therapeutic action against such diseases [37–40].

### 4.5. Direct Matrix Ranking

The output of a DMR showed the highest values (ranks) for a number of multipurpose medicinal plants of the study area such as* Morus alba*,* Dalbergia sissoo*,* Zizyphus jujuba*,* Withania coagulans*,* Tamarix aphylla*,* Fagonia cretica,* and* Nannorhops ritchieana*. The result indicates that these plants are exploited more for their nonmedicinal uses than for reported medicinal values. Overharvesting of multipurpose medicinal plant species for construction, fuel wood, fodder, and agricultural tools was found to be the responsible factors aggravating the depletion of the highly ranked species in the area.

### 4.6. Indigenous Knowledge

It was observed during research study that the knowledgeable women were more concentrated as compared to the men of this region of Pakistan. Generally, gender-based differences in medicinal plant knowledge can be derived from experience and degree of cultural contact with curative plants [41]. The study indicates that the aged people of the region have traditional knowledge about more numbers of medicinal plants as compared to younger people which might be due to their least interest. Hussain et al. [42] in South Waziristan and Parveen et al. [43] in the Thar Desert of India have also reported that people older than 35 years of age are more knowledgeable than the young ones on medicinal plants and their uses.

## 5. Conclusions

In conclusion, D. I. Khan has plenty of medicinal plants and the people of the region are highly dependent on these plants for medicinal and other ethnobotanical purposes. The people of the region have tremendous traditional knowledge regarding the utilization and preparation of various ethnomedicinal remedies. Moreover, they are using some medicinal plants for multipurpose and posing great pressures on certain medicinal plants like* Morus alba* and* Dalbergia sissoo*. Hence, natives should be educated regarding the sustainable usage of medicinal plants. The persistence of traditional knowledge is more among old age people; however, as a matter of concern, young people are taking less interest in such knowledge due to multiple reasons. As such, studies on the documentation of ethnomedicines may be extended to other areas for the protection of traditional knowledge. Further phytochemical analysis, pharmaceutical application, and clinical trials are therefore recommended in order to evaluate the authenticity of ethnomedicines to scientific standards.

## Figures and Tables

**Table 1:** Gender, age group, literacy level frequencies, and occupation of the interviewed people in the region.

	Total	Percentage
Gender		
Male	50	41.5
Female	70	58.5
Age groups		
21–29	2	1.6
30–39	8	6
40–49	22	18.3
50–59	21	18
60–69	23	19.1
70–79	27	22.5
80–89	17	14.1
Educational attainment		
Illiterate	63	52.5
Primary	31	25.8
Middle	16	13.3
Secondary	7	5.8
University	3	2.5
Occupation		
Females		
Housewives	63	90
Primary teacher	7	10
Males		
Shopkeepers	12	24
Farmers	22	44
Labour	8	16
Primary teachers	8	16

**Table 2:** Ethnomedicines of the study area.

Botanical name/voucher specimen number	Local name	Family	Habit/endemism	Status	Part used	Recipes	Dosage

*Acacia modesta *Wall. KUH-309	Kikar	Mimosaceae	Tree/nonendemic	Cultivated	Bark	Bark is boiled in water and extracted juice is used orally for kidney pain.	Mostly 2 or 3 times a day.

*Acacia nilotica (L.) *Delile. KUH-310	Kikar	Mimosaceae	Tree/nonendemic	Wild	Bark and flowers	Bark is boiled in water and the decoction is used dentally. Flowers are tied on affected area. Flowers are boiled in mustard oil; the oil is applied to head.	For dental purposes, it is used twice a day for 10 days, while for others it is used as needed.

*Albizia lebbeck *(L.) Benth. KUH-311	Sirsirin	Mimosaceae	Tree/nonendemic	Cultivated	Seeds	Seeds are ground to make powder and strained through fine cloth (having small pores). Equal quantities of the strained powder and sugar are mixed. This is an effective phytotherapy taken orally for asthma.	4 g of this mixed powder is used twice a day for a period of 40 days.

*Allium cepa *L. KUH-312	Piaz	Liliaceae	Herb/nonendemic	Cultivated	Bulb	Grind the bulb of onion and mix it with water and it is taken orally for vomiting.	One dose is enough for vomiting.

*Allium sativum *Linn. KUH-313	Garlic	Liliaceae	Herb/nonendemic	Cultivated	Bulb and leaves	Bulb pieces are regularly used for high blood pressure. Leaves and bulb are chewed for vomiting.	As needed.

*Aloe barbadensis*Mill. KUH-314	Kunwar	Liliaceae	Herb/nonendemic	Wild/cultivated	Pulp of Leaves	Two leaves are made spineless and each one is divided length-wise into 2 or 3 slices. These slices of leaves along with common salt are given orally to the animals. Pulp is directly applied on burned skin.	Usually 3 doses are given each after 48 hours and also depend on disease severity.

*Avena sativa *L. KUH-315	Jou	Poaceae	Herb/nonendemic	Cultivated	Fruit	Partly ripen fruit is ground and mixed with some sugar and cold water to make syrup and is taken orally as laxative.	As needed.

*Azadirachta indica *(L.) A. Juss. KUH-316	Neem	Meliaceae	Tree/nonendemic	Wild/cultivated	Leaves	3 gm of leaves is crushed and mixed with water and common salt to make syrup and used orally for curing jaundice.	Two spoons of syrup are used in the morning after breakfast and in the evening before meal.

*Beta vulgaris *Linn. KUH-317	Chukandar	Chenopodiaceae	Herb/nonendemic	Cultivated	Root	Powder is used orally to treat diabetes.	As needed.

*Calotropis *procera. Ait. f., Hort. KUH-318	Akra	Asclepiadaceae	Shrub/nonendemic	Wild	Latex and flowers	Plant latex is applied externally on the region of snake bite and 5–8 flowers are used at a time with water for intestinal pain.	2-3 times daily for 5 days for intestinal pain.

*Capparis desidua (Forsk.) Edgew. * KUH-319	Kareeta	Capparidaceae	Shrub/nonendemic	Wild	Fruit, Young shoots, and bark	Equal amounts of dried fruit and sugar are ground to make powder (*safoof*) and used orally for rheumatism. Young shoot is crushed and mixed with flour and given to animals. Bark is crushed and applied externally to the affected area for wound healing.	One teaspoon of powder is taken with a glass of water twice a day for a week for rheumatism.

*Capsicum annum *L. KUH-320	Green mirch	Solanaceae	Herb/nonendemic	Cultivated	Fruit	Fresh and unripe fruit is eaten for cancer.	As needed.

*Caralluma tuberculata *N Brown. KUH-321	Chunga	Apocynaceae	Herb/nonendemic	Wild	Whole plant	Cook it as simple vegetable for diabetics control and also it is eaten directly for many purposes.	As needed.

*Chenopodium album *L. KUH-322	Bathu	Chenopodiaceae	Herb/nonendemic	Wild	Leaves and root.	Extract the juice from their leaves which is taken orally as a laxative. Dried leaves paste is used for urinary pain.	As needed.

*Cicer arietinum *Linn. KUH-323	Channa	Papilionaceae	Herb/nonendemic	Cultivated	Fruits and seeds	25 gm of seed coat (testa) of roasted grams is soaked in 250 g of water at night and crushed in the morning and strained. The strained water is taken orally. This phytotherapy is considered to be useful for constipation.	As needed.

*Cichorium intybus L. *KUH-324	Kasni	Asteraceae	Herb/	Wild	Whole plant	Fresh plants are boiled and sugar is added to decoction to form syrup and used orally for stomach.	Syrup used twice a day for a period as needed.

*Cirsium arvense (L.) Scop. * KUH-325	Leh	Asteraceae	Herb/nonendemic	Wild	Leaves	Extract of leaves used for abdominal pain of cattle.	As needed.

*Citrullus colocynthis *(L.). KUH-326	Karthuma	Cucurbitaceae	Herb/nonendemic	Wild	Fruit	Crush the dried fruit and make *safoof* and use it orally for constipation.	As needed.

*Citrus medica *Linn. KUH-327	Nimbo	Rutaceae	Tree/nonendemic	Cultivated	Fruits and leaves	Fruit extract is mixed with water, salt, and sugar and used orally to control blood pressure and vomiting. Fruit extract is mixed with honey and fresh milk to make its paste and applied on face for pimples.	For skin the paste is used at night for one month; for other purposes, it is used as needed.

*Cleome brachycarpa *Vahl. KUH-328	Gandi booti	Capparidaceae	Herb/nonendemic	Wild	Whole plant	Plants dried under shade are ground to make powder. The pure powder is used orally for abdominal pain.	As needed.

*Convolvulus arvensis *L. KUH-329	Wanvehri	Convolvulaceae	Herb/nonendemic	Wild	Whole plant	5 kg plants, dried under shade, are mixed with 12 liters of water at night to make syrup and used orally for skin diseases.	In the morning one cup of this water is used on empty stomach or is used thrice a day for a period as needed.

*Cordia dichotoma *Forster.f. KUH-330	Lasora	Boraginaceae	Tree/nonendemic	Wild	Fruit	Eating of fruit, as needed, before meal is recommended for the treatment of masculine sexual weakness.	As needed.

*Coriandrum sativum *L. KUH-331	Dhania	Apiaceae	Herb/nonendemic	Cultivated	Leaves and fruits	About 50 g of dried fruit is boiled in one liter of water till half of the water is left. The decoction is used for asthma and cough. Fresh leaves are ground with mint to make powder and used orally for diuretic purposes.	Depends upon disease severity.

*Cucumis sativus*. Linn. KUH-332	Kheera	Cucurbitaceae	Herb/nonendemic	Cultivated	Fruits	During severe fever their pieces are rubbed on the sole of the feet and also good for digestion.	As required.

*Cuscuta reflexa *Roxb. KUH-333	Loot booti	Convolvulaceae	Herb/nonendemic	Wild	Whole plant	Plants are dried and burnt. The ash is applied to the affected area.	As needed.

*Cymbopogon jawarancu*sa (Jones) Schult. KUH-334	Khawi	Poaceae	Herb/nonendemic	Wild	Root	Upper parts of the roots are boiled in water. The water is strained and is given orally to the children along with sugar for dyspepsia and typhoid.	2-3 times daily.

*Daucus carota *L. KUH-335	Gajar	Apiaceae	Herb/nonendemic	Cultivated	Root	Edible portion increases sight vision.	As needed.

*Dalbergia sissoo *Roxb. KUH-336	Tali	Fabaceae	Tree/nonendemic	Cultivated	Leaves	70 g of young leaves of buds is crushed. One glass of water is added to it and strained. This is useful for piles, jaundice, and the feeling of hotness in sole of the feet.	The strained water is taken daily and continued for 10 days.

*Datura metel *L. KUH-337	Datura	Solanaceae	Herb/nonendemic	Wild	Whole plant	Roost their leaves and inhale their smoke for the treatment of asthma. Their seeds are used as expectorant, and do not eat it in excess amount; otherwise it will be lethal.	As needed.

*Eruca sativa *Mill. KUH-338	Usoo	Brassicaceae	Herb/nonendemic	Cultivated	Aerial parts	5 kg young branches along with leaves are boiled in 15 kg water and decoction is obtained. Then 5 kg millet flour is mixed with the decoction and is given orally to the horses.	As needed.

*Eucalyptus camaldulensis *Dehnh. KUH-339	Safeda	Myrtaceae	Tree/non endemic	Cultivated	Leaf and bark	Tender shoot and young leaves are crushed and their smell gives temporary relief in cough.	As needed.

*Eugenia jambolana *Linn. KUH-340	Jaman	Myrtaceae	Tree/nonendemic	Cultivated	Bark and fruit	Dry the nonedible portion of their fruit and then grind it and take 1-2 spoons daily for 3 days; it will stop the severe stomach problems, and also this powder is used for the treatment of diabetics.	Twice a day.

*Fagonia cretica L. *KUH-341	Dhaman	Zygophyllaceae	Shrub/nonendemic	Wild	Whole plant	Plant is crushed and decoction is taken with water for piles and urinary infection.	1 teaspoon of powder is taken with bread thrice a day.

*Ficus benghalensis *L. KUH-342	Bohir, bargad	Moraceae	Tree/nonendemic	Cultivated	Fruit	Fruit, dried under shade, is ground to form powder. This powder is used orally to treat abdominal pain.	Taken with water twice in day.

*Ficus carica *L. KUH-343	Anjeer	Moraceae	Tree/nonendemic	Cultivated	Fruit	2–4 figs (fruit) are soaked in water or milk at night and used in the morning on empty stomach. This is considered to be very effective for the treatment of piles. Fruit is used to remove kidney stone.	Used in the morning on empty stomach for 10 days.

*Ficus religiosa *L. KUH-344	Peepal	Moraceae	Tree/nonendemic	Cultivated	Fruit and bark	Burn the bark and make powder from this coal bark and take orally 5 grams of it with water for diarrhoea. Fruit is used for wound healing.	As needed.

*Foeniculum vulgare *Mill. KUH-345	Saunf	Apiaceae	Herb/nonendemic	Cultivated	Fruit	Equal quantities of fennel fruit, coriander fruit, and sugar are mixed and ground together to make powder and recommended as carminative. Fennel fruit, fresh mint leaves, and green tea are boiled used for vomiting.	The powder is used twice a day after meal as carminative and for vomiting and for menses pain the mixture is used twice a day.

*Grewia asiatica *L. KUH-346	Phalsa	Tiliaceae	Herb/nonendemic	Wild	Leaves, fruit, and bark	1 kg fruit is crushed with fingers in 1 liter of water and then strained. Sugar is added to the strained juice to make syrup. The syrup is taken orally for blood purifying, fever, and gastrointestinal disorders.	As needed.

*Helianthus annuus *L. KUH-347	Suraj mukhi	Asteraceae	Shrub/nonendemic	Cultivated	Seeds, leaves, and flowers	Leaves and seeds are crushed and used orally and dermally against fever and other purposes.	For 2 days.

*Heliotropium europaeum *L.KUH-348	Peepat Booti	Boraginaceae	Herb/nonendemic	Wild	Whole plant	The plant is crushed to make paste. The paste is applied as poultice to the affected area.	For 3 days.

*Jasminum grandiflorum *L. KUH-349	Cha mbeli	Oleaceae	Herb/nonendemic	Cultivated	Whole plant	Juice of whole plant is prepared and used orally against heart diseases and diabetes.	As needed.

*Lawsonia inermis *L. KUH-350	Mehndi	Lythraceae	Tree/nonendemic	Cultivated	Leaves	Leaves are crushed and paste is applied on soles and hairs.	As needed.

*Momordica charantia *Linn. KUH-351	Karela	Cucurbitaceae	Herb/ nonendemic	Cultivated	Leaf, flower, and fruits	Leaves are crushed and boiled and taken as tea; some salt is also mixed and used for skin diseases and blood purification. Fruit is used as vegetable and used for diabetics.	As needed.

*Mentha viridis *L. KUH-352	Podina	Lamiaceae	Herb/nonendemic	Cultivated	Leaves	Fresh leaves of mint, *niazbo*, fennel fruit, and green tea are boiled and used orally for vomiting and stomach disorders	As needed.

*Moringa oleifera *La m. KUH-353	Sohanjna	Moringaceae	Tree/nonendemic	Cultivated	Whole plant	Cut their root and boil it in water and after that add milk to this water and drink it which breaks the kidney stone.	As needed.

*Morus alba *L. KUH-354	Toot	Moraceae	Tree/nonendemic	Cultivated	Fruit	Eat their fruits which provide the energy to the heart.	As needed.

*Nannorrhops ritchieana *Griff. KUH-355	Mazri	Palmae	Shrub/nonendemic	Wild/cultivated	Leaves	Mostly their leaves are boiled and then this juice is used orally for carminative and veterinary treatment.	

*Ocimum basilicum *L. KUH-356	Niazbo	Lamiaceae	Herb/nonendemic	Cultivated	Seeds and leaves	Fresh leaves of mint, niazbo, fennel fruit, and green tea are boiled and used for gastrointestinal and respiratory infections.	As needed.

*Oxalis corniculata *L.KUH-357	Khatti boti	Oxalidaceae	Herb/nonendemic	Wild	Whole plant	Extract of whole plant is used orally for blood purification.	As needed.

*Peganum harmala *L.KUH-358	Harmal	Zygophyllaceae	Herb/nonendemic	Wild	Leaves and seeds	A small quantity of harmala seeds along with small quantity of table salt is taken with water for a few days as expectorant.	Dose depends on disease severity.

*Phoenix dactylifera *L. KUH-359	Khajoor	Palmae	Tree/nonendemic	Cultivated	Leaflets (spines)	The lowest leaflets (spines) of compound leaf are crushed and boiled. The strained water is taken orally for general pain.	As needed.

*Plantago ovata *Forsk. KUH-360	Ispaghula	Plantaginaceae	Shrub/nonendemic	Wild	Seeds	12 gm seeds are taken with milk at night for constipation. 12 gm seeds, sugar, and 1 glass of water are mixed and shaken well to cure jaundice.	Twice a day.

*Polygonum barbatum *L. KUH-361	Karaveera	Polygonaceae	Herb/nonendemic	Wild	Whole plant	Plants are crushed to form paste; the paste is used as poultice on the affected area daily for 3 days.	For a period of three days.

*Portulaca oleracea *L. KUH-362	Lunrak	Portulacaceae	Herb/nonendemic	Wild	Seeds	Equal amounts of seeds of *Portulaca*, *coriander*, *Argyreia speciosa*, and table sugar are ground to make powder (*safoof*). It is an effective traditional phytotherapy used for night emission.	10 gm powder is taken with water twice a day.

*Punica granatum *L. KUH-363	Anar	Punicaceae	Tree/nonendemic	Cultivated	Fruit	Crush the dried fruit and mix this *safoof* with water and it is given orally to children.	Mostly twice a day.

*Raphanus sativus *L. KUH-364	Mooli	Brassicaceae	Herb/nonendemic	Cultivated	Roots and edible parts	Paste of root is formed and used for skin infections.	Two times a day.

*Ricinus communis *L. KUH-365	Hernoli	Euphorbiaceae	Shrub/nonendemic	Wild	Leaves and fruit	Heat the leaves and fruits and then they release the oil which is leaped on the desired place or organs where pain is felt.	As needed.

*Rosa indica *Lindl, Ros. Monogr. KUH-366	Gulab	Rosaceae	Shrub/nonendemic	Cultivated	Flower	Fresh petals are mixed with sugar to make *gulkand *and kept in bottle and used for stomach disorders.	As needed.

*Saccharum benghalensis * Retz. KUH-367	Kana	Poaceae	Herb/nonendemic	Wild	Leaves	Ash of the leaves is mixed with water, and after an hour it will settle down in the bottom. The strained water is given to the animals suffering from “urine retention” disease.	As needed.

*Salvadora oleoides Decne.* KUH-368	Jal	Salvadoraceae	Tree/nonendemic	Wild	Fruit	250 gm of fruits is placed in a clayey pot and its mouth is closed in order to prevent the entrance of water in the pot. The pot is placed in a bucket of water for a night. The fruit is used in the morning on empty stomach. Eating of fruit of water melon before or after the eating of Salvadoran fruit is useful.	For one week.

*Salvadora persica *L. KUH-369	Peelu	Salvadoraceae	Shrub/nonendemic	Wild	Bark	Bark is boiled in water, taken as tea, and commonly used as a purifying agent.	As needed.

*Solanum surattense* Burm.f. KUH-370	Kandari	Solanaceae	Herb/nonendemic	Wild	Whole plant	The powder of dried fruits is taken with water for a period to treat piles. Fresh plants are boiled in 3 times more water. The water is filtered and mixed with sugar to form syrup and used for eczema and blood purification.	Syrup is used twice a day as needed for a period of for 2 months for treatment.

*Solanum nigrum *L. KUH-371	Makko	Solanaceae	Herb/nonendemic	Wild	Leaf and fruit	Ripe fruits are directly given orally for constipation; plant paste is formed and applied dermally for headaches and joint pain. Plant juice is used for dysentery and fever.	As needed.

*Tamarix aphylla *(L.) Karst. KUH-372	Khagal	Tamaricaceae	Shrub/nonendemic	Cultivated	Bark, leaves, and twigs	Ash of the leaves is mixed with water; after half an hour the water is strained (filtered) and boiled. After boiling, the water is evaporated and the salt is left behind. Then 1 gm salt is taken with *Sharbat-e-Bazoori*. It is a useful traditional phytotherapy for jaundice. Leaves are boiled in water. The water is strained and the hot leaves are tied on the affected area daily. This phytotherapy is used for the treatment of wound.	Twice a day for a period as needed for jaundice; for wound healing, it is used for one week.

*Thuja occidentalis *L. KUH-373	Thuja	Cupressaceae	Tree/nonendemic	Cultivated	Leaves	Boil their leaves in the water and then wash the mouth with this water; it provides rapid relief in dental pain. Fresh leaves are burned and their smoke decreases temperature in fever.	As needed.

*Tribulus terrestris *L. KUH-374	Bhakra, Gokhru	Zygophyllaceae	Herb/nonendemic	Wild	Fruit	The fruit is crushed and dried. Sugar, as needed, is mixed with the dried powder and is used orally for urinary infection.	Four times in a day for 3 months.

*Viola stocksii Boiss. *KUH-375	Makhanr booti	Violaceae	Herb/nonendemic	Wild	Whole plant	The whole plant, along with seeds, is ground to make powder and used for sexual purpose.	2 g powder is used with 1 teaspoon butter early in the morning on empty stomach as needed.

*Withania coagulan*s (Stocks) Dunal. KUH-376	Akri	Solanaceae	Herb/nonendemic	Wild	Fruit	Five to six dried fruits are soaked in 2-3 cups of water at night; in the morning the soaked fruits are squeezed and the water is strained and used for blood purification. One dried fruit is kept in teeth keeping pain. Five to six fruits are taken with water like tablet for abdominal pain.	One cup of water is used on empty stomach for blood purification. And for other purposes, it depends upon disease severity.

*Zea mays *Linn. KUH-377	Makkai	Poaceae	Herb/nonendemic	Cultivated	Fruit	Dry fruit is crushed and made into flour which is used for digestion.	As needed.

*Ziziphus jujuba *Mill. KUH-378	Ber	Rhamnaceae	Tree/nonendemic	Wild/cultivated	Leaves and fruits	Eat their fruit which is helpful in treatment of diarrhoea as well as in blood purification. Paste of leaves is used for hair growth.	As needed.


**Table 3:** Informant consensus factor (F_IC_) for different ailment categories.

Use categories	Plant species	Number of taxa (N_t_)	Number of use reports (N_ur_)	*F*_IC_
Gastrointestinal	*Avena sativa*. (4), *Calotropis procera. *(6), *Chenopodium album. *(3), *Cicer arietinum*. (2), *Cichorium intybus. *(5), *Citrullus colocynthis. *(9), *Cleome brachycarpa. *(5), *Cucumis sativus*. (7), *Cymbopogon jawarancusa. *(6), *Eugenia jambolana. *(12), *Ficus benghalensis*. (4), *Ficus religiosa*. (4), *Foeniculum vulgare*. (8), *Grewia asiatica*. (5), *Helianthus annuus. *(6), *Mentha viridis*. (5), *Nannorrhops ritchiana.* (4), *Ocimum basilicum*. (5), *Plantago ovata. *(7), *Punica granatum*. (6), *Raphanus sativus, Solanum nigrum *(4), *Zea mays*. (3), *Ziziphus jujuba*. (2).	24	122	0.80

Respiratory	*Albizia lebbeck*. (3), *Coriandrum sativum*. (3), *Datura metel. *(2), *Eucalyptus camaldulensis. *(5), *Ocimum basilicum. *(1), *Peganum harmala. *(6), *Salvadora oleoides*. (3).	7	23	0.72

Skin infections	*Convolvulus arvensis. *(2), *Jasminum grandiflorum. *(1), *Momordica charantia. *(4), *Raphanus sativus. *(2), *Solanum surattense*. (7).	5	16	0.73

Fever	*Cucumis sativus*. (1), *Cymbopogon jawarancusa.* (2), *Grewia asiatica. *(5), *Helianthus annuus. *(2), *Solanum nigrum. *(1), *Thuja occidentalis. *(3).	6	14	0.61

Wound healing	*Capparis decidua. *(2), *Cuscuta reflexa. (1), Ficus religiosa. (1), Tamarix aphylla*. (2).	4	6	0.41

Cardiovascular	*Jasminum grandiflorum. *(3), *Morus alba*. (2), *Raphanus sativus*. (2).	3	7	0.66

Jaundice	*Azadirachta indica. *(3), *Dalbergia sissoo*. (1), *Plantago ovata. *(4), *Tamarix aphylla*. (2).	4	10	0.66

Rheumatism	*Capparis decidua. *(1), *Polygonum barbatum. *(2).	2	3	0.50

Vomiting	*Allium cepa*. (2), *Allium sativum*. (1), *Citrus medica*. (3), *Foeniculum vulgare*. (5), Mentha *viridis*. (2).	5	13	0.66

Kidney problems	*Acacia modesta. *(4), *Chenopodium album*. (5), *Coriandrum sativum*. (2), *Fagonia cretica. *(6), *Ficus carica*. (3), *Helianthus annuus*. (8), *Moringa oleifera*. (4), *Tribulus terrestris. *(5).	8	37	0.80

**Table 4:** Average direct matrix ranking (DMR) score of fifteen key informants for ten medicinal plants species.

Use diversity	*C. desidua *	*Z. jujuba *	*D. sissoo *	*T. aphylla *	*F. cretica *	*W. coagulans *	*M. alba *	*N. retichiana *	Total	Rank
Fuel	3	4	4	4	2	3	4	3	**27**	1
Medicinal	3	3	3	4	4	5	3	2	**27**	1
Fodder	3	3	3	4	2	4	4	3	**26**	2
Agricultural tools	2	3	5	3	2	1	3	2	**21**	3
Construction	1	3	5	3	1	2	3	3	**21**	3
Food	1	4	1	1	1	2	4	1	**15**	4
Total	**13**	**20**	**21**	**19**	**12**	**17**	**21**	**14**		
Rank	6	2	1	3	7	4	1	5		

Based on use criteria (5 = best; 4 = very good; 3 = good; 2 = less used; 1 = least used; 0 = no value).

## Questionnaire of Ethnobotanical Data Collection**

* Informants' Consent for the Participation in the Study.*

I…(name of informant) hereby give my full consent and conscious to participate in this study and declare that to the best of my knowledge the information that I have provided is true, accurate, and complete.

Date…(signature/thumb impression of informant).

* Informants' Details.*

Name.
Gender.
Age.
Ethnicity.
Occupation.
Education.
Location/residence.

* Data about Medicinal Plant and Its Use.*

Number of plants known.
Names of plants (local names).
Plant part used.
Cultivated/wild.
Name of disease(s) treated.
Name of plants used for specific disease categories.
Method of crude drug preparation.
Use of single or mixture of plants.
Mode of administration.
Dosage.

* Informant Consensus Factor.*

Name of plants used against disease category 1.
Name of plants used against disease category 2.
Name of plants used against disease category 3.
Name of plants used against disease category 4.
Name of plants used against disease category 5.
Name of plants used against disease category 6.
Name of plants used against disease category 7.
Name of plants used against disease category 8.
Name of plants used against disease category 9.
Name of plants used against disease category 10.

* Direct Matrix Ranking.*

Number of plants having NTFPs uses.
Name of plants (local names).
NTFPs uses.

* Remarks.*

Plant identified as——(botanical name and family).

Signature of researcher
